# The Remedial Potential of Lycopene in Pancreatitis through Regulation of Autophagy

**DOI:** 10.3390/ijms21165775

**Published:** 2020-08-12

**Authors:** Suyun Choi, Hyeyoung Kim

**Affiliations:** Department of Food and Nutrition, Brain Korea 21 PLUS Project, College of Human Ecology, Yonsei University, Seoul 03722, Korea; chltndus0517@naver.com

**Keywords:** autophagy, inflammatory responses, lycopene, oxidative stress, pancreatitis

## Abstract

Autophagy is an evolutionarily conserved process that degrades damaged organelles and recycles macromolecules to support cell survival. However, in certain disease states, dysregulated autophagy can play an important role in cell death. In pancreatitis, the accumulation of autophagic vacuoles and damaged mitochondria and premature activation of trypsinogen are shown in pancreatic acinar cells (PACs), which are the hallmarks of impaired autophagy. Oxidative stress mediates inflammatory signaling and cytokine expression in PACs, and it also causes mitochondrial dysfunction and dysregulated autophagy. Thus, oxidative stress may be a mediator for autophagic impairment in pancreatitis. Lycopene is a natural pigment that contributes to the red color of fruits and vegetables. Due to its antioxidant activity, it inhibited oxidative stress-induced expression of cytokines in experimental models of acute pancreatitis. Lycopene reduces cell death through the activation of 5′-AMP-activated protein kinase-dependent autophagy in certain cells. Therefore, lycopene may ameliorate pancreatitis by preventing oxidative stress-induced impairment of autophagy and/or by directly activating autophagy in PACs.

## 1. Introduction

Pancreatitis is a gastrointestinal disorder with considerable morbidity and mortality [1]. Acute pancreatitis, recurrent acute pancreatitis, and chronic pancreatitis form a disease continuum [2]. Some factors causing these diseases include alcohol, bile acids, and ductal obstruction, where they induce the inflammatory response that promotes their pathogenesis [3]. Common cellular events underlying the pathogenesis of acute pancreatitis include increased oxidative stress, premature intra-acinar trypsinogen activation, activated calcium signaling, mitochondrial dysfunction, endoplasmic reticulum (ER) stress, and autophagy impairment [3,4,5].

Autophagy is a highly conserved cellular recycling process that involves lysosomal degradation of cellular substrates and the release of the breakdown products back into the cytosol [6,7,8]. After being released into the cytosol, these products are reused to support biosynthesis or are catabolized for energy generation [6,7,8].

Autophagy occurs at a basal rate in most cells, where it acts as a quality control mechanism to eliminate protein aggregates and damaged or unneeded organelles. Equally important, autophagy constitutes a major protective mechanism that allows cells to survive and adapt to fluctuations in external conditions. In particular, nutrient deprivation is one of the strongest inducers of physiological autophagy; it generates/recycles cellular components (e.g., amino acids) vital for cell survival [9,10].

There are three major types of autophagy that occur in mammalian cells: chaperone-mediated autophagy, microautophagy, and macroautophagy [6,7,8]. The major form of autophagy is macroautophagy. It involves the sequestration of cytosolic components, such as long-lived proteins, lipids, and organelles, by double-membrane vacuoles known as autophagosomes [6,7,8]. Autophagosomes generate autolysosomes after fusing with endosomal and lysosomal membranes. The sequestrated cytosolic components are eventually degraded by lysosomal hydrolases and recycled back into the cytosol [6,7,8]. Macroautophagy, which is the major and most prevalent type of autophagy, is also the best-characterized type defining the general term “autophagy”.

Autophagosome formation is a complex process [11]. It begins with the formation of a phagophore that elongates to engulf parts of the cytoplasm (including organelles such as mitochondria) and closes to form the mature autophagosome. This process is controlled by a series of evolutionarily conserved autophagy-related genes (ATGs), as well as lipid kinases, such as the class III phosphatidylinositol 3-kinase vacuolar protein sorting 34 (VPS34) [12]. ATG proteins control individual steps of the autophagosome formation process and are required for the assembly of the isolation membrane. A product of the ATG8 gene, microtubule-associated 1A/1B-light chain 3 (LC3) protein, is necessary for phagophore closure. Its cytosolic LC3-I form is modified (via lipidation) to become LC3-II, which specifically translocates to the autophagosomal membrane [13].

Impaired autophagy has been proposed to be associated with pancreatitis. Accumulation of autophagic vacuoles has been shown in acinar cells in animal models [14,15] and in human disease [16,17,18,19]. The accumulation of large vacuoles was accompanied by increased pancreatic levels of LC3-II and was observed in all experimental models [15]. Importantly, and in contrast to starvation, pancreatitis greatly decreases autophagic efficiency, which is manifested by a decreased rate of long-lived protein degradation and an increased level of p62 [20].

The presence of LC3-II, a marker of mature autophagosomes, on autophagic vacuoles has been shown in acute pancreatitis rodent models treated with the cholecystokinin (CCK) analog cerulein. The autophagic vacuoles that accumulated in acinar cells in experimental pancreatitis were predominantly large autolysosomes, indicating inefficient lysosomal degradation [14,15]. Disruption of the genes encoding ATG7, or lysosomal associated membrane protein (LAMP) 2, impair autophagy in the pancreas and induce spontaneous pancreatitis, coupled with inflammation and pancreatic atrophy in experimental animals [16,21,22]. In patients, pancreatitis is associated with a decrease in pancreatic levels of LAMPs [15] and an accumulation of p62 and LC3-II in acinar cells [9,23]. These changes might be targeted for the treatment of pancreatitis.

In experimental pancreatitis, intracellular Ca^2+^ overload induces activation of NADPH oxidase to produce reactive oxygen species (ROS) in pancreatic acinar cells (PACs) [24]. Moreover, increased intracellular Ca^2+^ directly damages mitochondria via mitochondrial matrix Ca^2+^ overload and ROS accumulation in PACs [25,26]. In acute pancreatitis, ROS are produced in PACs exposed to alcohol and high levels of cytokines [27,28,29]. Thus, reducing ROS levels has been suggested for preventing the development of acute pancreatitis.

Autophagy can protect cells from ROS-mediated damage through the elimination of damaged organelles [30,31]. However, if the amount of ROS overrides the capacity of basal autophagy, or if the autophagic mechanism is impaired, oxidative stress can exacerbate pancreatitis symptoms by injuring cellular components and promoting inflammatory responses [30,31].

As mentioned above, autophagy is impaired in pancreatitis, with a lower degradation rate of long-lived proteins, premature activation of trypsinogen, and accumulation of autophagic vacuoles in PACs. Thus, reducing oxidative stress may protect PACs against autophagic impairment.

Lycopene, a non-provitamin A carotenoid, is the most prevalent carotenoid in the human diet [32]. Lycopene is an efficient ^1^O_2_ quencher, and its ability to trap ^1^O_2_ is better than those of vitamin E and thiols, as assessed by determining the quenching ability of ^1^O_2_ generated by the thermodissociation of the endoperoxide of 3,3′-(1,4-naphthylidene) dipropionate [33]. Recently, Milani et al. [34] demonstrated that the beneficial effects of carotenoids in health and in decreasing the risk of certain diseases are attributed to the major carotenoids β-carotene, lycopene, lutein, and zeaxanthin due to their antioxidant effects. The antioxidant activity of lycopene is mainly dependent on its O^2−^ and -OH scavenging properties [35].

Carotenoid uptake through the brush border of enterocytes has been considered to occur by passive diffusion [36]. Recent studies have shown that the absorption of carotenoids may be facilitated by other transporters. Caco-2 cells demonstrated that scavenger receptor class B type-1 (SR-B1) plays an important role in the cellular uptake of lutein [37]. The xanthophylls lutein and zeaxanthin were taken up by cells up to 2-fold higher than beta-β-carotene uptake in an SR-B1-dependent mechanism in adult retinal pigment epithelial cells [38]. Shyma et al. [39] determined whether three scavenger receptor class B (SRB) proteins, SR-B1, SR-B2, and cluster determinant 36 (CD36), bind carotenoids in macular tissues. They found that lutein uptake was enhanced in the presence of low-density lipoprotein and is mediated by SR-B1 and CD36. SR-B1, SR-B2, and CD36 were able to take up significant amounts of zeaxanthin as well as mesozeaxanthin, and their uptake was increased in the presence of high-density lipoprotein. Lycopene uptake is facilitated by SR-B1 in human intestinal cells [40] and CD36 in adipocytes [41]. Further studies should be performed to determine the transporters responsible for stimulating lycopene uptake in various cells for us to understand the protective mechanism of lycopene against the development of certain diseases, including pancreatitis.

Humans absorb a significant portion of intact lycopene directly, and it circulates through and accumulates in their plasma, liver, and peripheral tissues [42]. Lycopene was found to be cleaved by a purified recombinant murine and ferret intestinal β-carotene 15,15′-oxygenase (β-carotene oxygenase 1; BCO1) with low activity, as compared to β-carotene cleavage by BCO1 [43]. It may be because the main role of BCO1 is to symmetrically cleave β-carotene [44]. High levels of BCO1 mRNA were observed along the whole intestinal tract, in the liver, and in the kidney, whereas lower levels were present in the prostate, testis, ovary, and skeletal muscle. Current data suggest that the human BCO enzyme, in addition to its well-established role in the digestive system, may also play a role in peripheral vitamin A synthesis from provitamin A carotenoids [44]. Moreover, β-carotene 9′,10′-oxygenase (β-carotene oxygenase 2; BCO2) can catalyze the eccentric cleavage of lycopene to form apo-10′-lycopenoids [45]. Von Lintig and Vogt cloned and identified BCO1 from *Drosophila melanogaster* [46]. Wyss et al. [47] cloned the eukaryotic BCO1, which symmetrically cleaves β-carotene at the 15,15′-double bond. Kiefer et al. [48] identified and characterized BCO2, and demonstrated that both symmetric and asymmetric cleavage pathways exist for carotenes, where they also revealed a greater complexity of carotene metabolism in vertebrates. Von Lintig’s and Wyss’ teams developed two knockout mouse models lacking BCO1 and BCO2, respectively [49,50]. The most striking characteristic of both mouse models was their ability to accumulate carotenoids, similar to how it occurs in humans.

Coonel et al. [51] summarized that BCO1^−/−^ mice accumulate β-carotene, while BCO2^−/−^ mice accumulate xanthophylls (such as lutein and zeaxanthin), as well as lycopene. While BCO1 is a cytosolic enzyme, BCO2 is present in the inner mitochondrial membrane, where it protects this organelle against carotenoid accumulation. In addition, the exposure of either lycopene or its BCO2-mediated cleavage product, apo-10′-lycopenoic acid, attenuates the expression of proinflammatory cytokines in cultured adipocytes. Tan et al. [52] showed that serum lycopene concentrations were higher in lycopene- and tomato-fed BCO2^-/-^ mice compared with wild-type mice. Ford et al. [53] used female BCO1^-/-^ and BCO2^-/-^ mice fed with lycopene or tomato-containing diets for 30 days to determine the interactions between diet and genotype on carotenoid accumulation and lipid parameters. They found that BCO1^-/-^ mice had higher levels of insulin and hepatic lipidosis, but lower levels of serum cholesterol. BCO2^-/-^ mice had increased tissue lycopene and phytofluene accumulation, reduced insulin-like growth factor 1 levels and cholesterol levels, but elevated liver lipids and cholesterol, compared with wild-type mice.

Therefore, it is important to determine whether the effect of lycopene on various cellular functions and signaling pathways is the result of the action of intact lycopene or its derivatives. Since rodent models do not accumulate carotenoids, including lycopene, mutant animal models that are BCO1^-/-^ and BCO2^-/-^ may be useful to determine the biological activity as well as the interaction of lycopene with lipid mediators in the pathogenesis of pancreatitis.

Regarding its effect on autophagy, lycopene protects against apoptosis in oxidative stress-induced H9C2 myocardioblast cells through increased autophagy [54]. Previously, we showed that lycopene reduces ROS-mediated cytokine expression in cerulein pancreatitis in vitro [55]. Lycopene inhibits oxidative stress and activates autophagy. Therefore, lycopene has the potential to prevent autophagy impairment, which is shown in pancreatitis by reducing ROS-mediated Ca overload, mitochondrial dysfunction, premature activation of trypsinogen, and by preventing cell death of PACs.

Here, the role of autophagy in pancreatitis and the possible mechanisms by which lycopene prevents pancreatitis via the regulation of oxidative stress-associated autophagy impairment are discussed.

## 2. Autophagy Impairment and Pancreatitis

### 2.1. Steps in the Autophagy Process and Related Cellular Machinery

Autophagy occurs via a series of steps consisting of autophagosome formation, cargo selection, autophagolysosome formation, and autophagolysosome degradation [6,7]. In mammalian cells, autophagosome formation can be initiated at various membrane sites: throughout the ER, Golgi apparatus, mitochondria, recycling endosomes, and the plasma membrane [6,7]. Induction of autophagosome formation is regulated by a complex of proteins, including the Unc-51-like kinase (ULK) protein family members ULK1 or ULK2, ATG13, and the RB1-inducible coiled-coil protein 1 (RB1CC1/FIP200). Under nutrient-rich conditions, the ULK1/2–ATG13–RB1CC1 complex is associated with the mammalian target of rapamycin complex 1 (mTORC1). mTORC1 phosphorylates several sites in the protein complex, thus maintaining the induction complex in an inactive state [6,7]. However, under nutrient-poor conditions, mTORC1 dissociates, and the ensuing activation of the complex by dephosphorylation induces autophagy.

The second stage of autophagosome formation is nucleation, and it is mediated by the ATG14-containing class III phosphatidylinositol 3-kinase (PtdIns3KC3) complex [1,2]. This protein complex consists of VPS34, p150, and Beclin-1. Regulation of the PtdIns3KC3 complex typically occurs in a Beclin-1-dependent manner, in which B-cell lymphoma 2 protein (Bcl-2) interacts with Beclin-1, preventing its association with PtdInsK3C3, inhibiting autophagy [6,7].

After the ULK1/2–ATG13–RB1CC1 complex recruits VPS34, p150, and Beclin-1, the elongation stage begins, and an expanded membrane known as the phagophore is formed [6,7]. During elongation, the participation of the complex of ATG12, ATG5, and ATG16 proteins is required to ensure proper phagophore curvature [8]. To conjugate ATG12 and ATG5, the autophagy-related proteins ATG7 and ATG10 act as E1- and E2-like enzymes, respectively. The binding of ATG15 to ATG5 then follows. The elongation stage also requires the participation of a system involving LC3, which elongates and seals the phagophore to form the autophagosome structure [6,7]. LC3 is processed by the autophagy-related 4A cysteine protease (AGT4) to form LC3-I, which in turn, is transferred to the E2-like enzyme ATG3 by ATG7. Lipidation of cytosolic LC3-I with phosphatidylethanolamine to generate LC3-II is orchestrated by the ATG12–ATG5–ATG16 complex. LC3-II exclusively localizes to the autophagosomal membranes, where it remains until its fusion with the lysosome.

During the final stage of autophagy, the single membrane autolysosome is formed, and the cellular components contained within it are degraded [6,7]. The formation of the autolysosome requires soluble N-ethylmaleimide-sensitive factor attachment protein receptor (SNARE)-mediated fusion of the autophagosome directly with the lysosome or following the fusion of the autophagosome with an endosome. Degradation of the cellular components is catalyzed by lysosomal acid hydrolases. The lysosomal membrane protein vacuolar ATPase (V-ATPase) and lysosomal-associated membrane proteins 1 (LAMP-1) and 2 (LAMP-2) also play important roles in autophagy.

### 2.2. Cell Signaling Pathways Regulating Autophagy

The main signaling pathway regulating autophagy senses nutrient status; however, autophagy is also regulated by other mechanisms, including hormone sensing and energy sensing, as well as the cellular stress response and the response to pathogen infection.

One of the major negative regulators of autophagy is mTORC1, which senses nutrient signaling such as the physiological amino acid levels [6]. It has been proposed that in mammalian cells, the Ras-related small GTPase Rag proteins translocate mTORC1 to interact with Rheb (a Ras homolog enriched in brain), the mTORC1 activator, in response to amino acid levels [56,57]. VPS34, a component of PtdIns3KC3, is also thought to function as an amino acid sensor that activates mTORC1 to inhibit autophagy [58].

The enzyme AMPK (5’-AMP-activated protein kinase) is the main sensor of cellular ATP levels. Activated by liver kinase B1 (LKB1) in response to a decreased ATP/AMP ratio, AMPK phosphorylates and activates the tuberous sclerosis 1/2 (TSC1/2) complex, resulting in the inhibition of the mTORC1 activator, Rheb [59]. AMPK can also promote autophagy induction by activating p27^kip1^ [60]. In addition, AMPK can directly activate ULK1 via Ser317 and Ser777 phosphorylation, thereby promoting autophagy [61], whereas mTORC1 phosphorylates ULK1 Ser757, thereby blocking AMPK-mediated ULK1 activation [61]. Therefore, autophagy is inhibited through mTORC1 activation when the energy source is sufficient but is induced by energy deprivation through AMPK signaling.

In addition to nutrient starvation-induced autophagy, the absence of growth factors, including insulin, can also induce autophagy [62]. The hormone-mediated pathway includes the regulation of mTORC1. Following insulin receptor activation, IRS (insulin receptor substrate) 1 and 2 are recruited to create a scaffold for the activation of the adaptor protein class I PtdInsK. PIP3 (phosphatidylinositol (3,4,5)-triphosphate) generated by the class I PtdInsK recruits protein kinase B (PKB)/Akt and phosphoinositide-dependent protein kinase 1 (PDK1) [63]. PKB/Akt is phosphorylated and activated by PDK1, which subsequently phosphorylates TSC2 (tuberous sclerosis complex), preventing TSC2 interacting with TSC1 to form the TSC1/2 complex [64]. Since the TSC1/2 complex inhibits Rheb activation, active PDK/Akt allows Rheb to be in the active GTP-bound form, which is responsible for mTORC1 activation [65,66].

Functioning in the growth factor-sensing system, receptor tyrosine kinase-activated Ras signaling plays a role in autophagy by transducing the signal to other effectors, including class I PtdIns3K and Raf-1 [67]. Raf-1 is a nutrient sensor that responds to amino acid deprivation by inducing the mitogen-activated protein kinase (MAPK) pathway to downregulate mTORC1 activity [68]. Ras-activated Raf-1 activates mitogen-activated protein kinase kinase (MAPKK/MEK) 1/2, which, in turn, activates extracellular signal-regulated kinase (ERK) 1/2. Therefore, it was concluded that the two axes mediated by Ras are on opposite sides in regulating autophagy.

The MAPK family kinase ERK, c-Jun NH2-terminal kinase (JNK), and p38 MAPK regulate autophagy. In MAPK/JNK signaling, JNK1 phosphorylates Bcl-2, resulting in the dissociation of Bcl-2 from Beclin-1 [69]. In addition, JNK phosphorylates and activates c-Jun/c-Fos, which enhances the transcriptional activity of Beclin-1 [70]. JNK also activates forkhead box O (FoxO), a transcription factor that regulates numerous ATG genes [71].

The kinase p38 MAPK phosphorylates and thereby inactivates ULK1, which, in turn, results in autophagy inhibition [72]. p38 MAPK also phosphorylates glycogen synthase kinase 3β (GSK3β), which leads to AMPK activation and mTORC1 inactivation [73]. On the other hand, substantial evidence indicates that p38 MAPK mediates autophagy inhibition via ATG5 phosphorylation, and suggests the dual role of p38 MAPK in regulating autophagy [74].

ER stress is caused by various stimuli, including excessive protein aggregation, glucose deprivation, hypoxia, and oxidative stress [75]. The ER serves as a major Ca^2+^ reservoir, and ER stress leads to the release of luminal Ca^2+^ to the cytosol [76]. Besides ER stress, other extrinsic stimuli and different Ca^2+^ sources (including lysosomes) can influence cytosolic Ca^2+^ levels [77]. Increased Ca^2+^ levels result in the activation of calcium-activated calmodulin-dependent kinase kinase-β (CaMKKβ) [7]. CaMMKβ is responsible for the activation of AMPK and autophagy induction [64]. Additionally, an elevated Ca^2+^ level promotes phosphorylation and activation of protein kinase Cθ (PKCθ). Active PKCθ is required for the conversion of LC3-I to LC3-II, which is essential for the elongation of the autophagosome to occur [78].

### 2.3. Autophagy and Pancreatitis

During normal pancreatic function, inactive precursors (zymogens) of pancreatic digestive enzymes are packaged into exocrine granules (zymogen granules) in pancreatic acinar cells for secretion into the duodenal lumen. There, the trypsin zymogen trypsinogen is converted to the active protease trypsin, which functions to convert other digestive hydrolase zymogens to their active forms. During acute pancreatitis, zymogens are prematurely activated within the acinar cells, thereby causing cell damage and cell death via self-digestion [79].

During pancreatitis in humans or in experimental models, acinar cells accumulate large cytoplasmic vacuoles [14,15,80], predominately autolysosomes [22]. The accumulation of these large vesicles indicates impaired autophagic flux, consistent with increased levels of LC3-II and p62, and decreased rates of degradation of long-lived proteins [20,81]. Reduced autophagic flux manifests in the accumulation of autolysosomes containing partially digested cargo [14] owing to decreased levels of lysosomal hydrolase activity [82]. Notable among these hydrolases are the cathepsins, cathepsin B (CatB) and cathepsin L (CatL), whose respective functions are to convert trypsinogen to trypsin and to degrade both trypsin and trypsinogen [14]. It has been hypothesized that the imbalance between CatB and CatL activities underlies the accumulation of trypsin [77,78]. Trypsin accumulates in acinar cells as a result of deficient autophagic protein degradation, and impaired lysosomal cathepsin processing is responsible for trypsin accumulation and the development of pancreatitis [83,84].

### 2.4. Factors Related to Impaired Autophagy in Pancreatitis

#### 2.4.1. Oxidative Stress

Molecules such as ROS have been considered as early inducers of autophagy upon nutrient deprivation [85]. O_2_^−^ is the primary ROS involved in autophagy induced by glucose, glutamine, pyruvate, or serum deprivation [86], while H_2_O_2_ is the molecule produced immediately after starvation [87]. ROS produced by NADPH oxidase in macrophages upon bacterial infection recruit microtubule-associated protein light chain 3 (LC3) on phagosomes that are degraded by autophagy [88]. In response to nutrient deprivation, ATP demand causes mitochondrial overburden, resulting in an increased electron leakage and ROS production [89]. ROS also sequesters mTORC1, which is a negative modulator of autophagy [90]. Upon nutrient deprivation, ROS positively regulate autophagy through the activation of AMPK and oxidation of Arg4, which leads to the inactivation of the delipidating activity of LC3 and the accumulation of proautophagic LC3-II [91].

Even though ROS induce autophagy upon starvation and stress as a salvaging process, high levels of ROS mediate autophagy impairment in disease states such as nicotine-induced senescence of murine lungs [92], lung epithelial cells exposed to graphite carbon nanofibers [93], and diabetes mellitus [94]. ROS induced by bile acids cause apoptosis, with impaired production of ATP and mitochondrial dysfunction [95]. The mechanisms that determine whether ROS cause autophagy impairment in pancreatitis have not yet been established. However, both ROS and impaired autophagy are shown in acute pancreatitis [69,70,71]. Therefore, we can postulate the relationship of ROS to autophagy impairment in the development of acute pancreatitis.

#### 2.4.2. Ca^2+^ Overload

The primary initiating event in pancreatitis is the excessive rise in the concentration of cytosolic Ca^2+^ in pancreatic acinar cells [96]. Environmental stressors and genetic mutation increase the permeability of cellular membranes, and leaky membranes induce excessive Ca^2+^ influx into the cytosol [97]. Elevated Ca^2+^ levels impact signaling pathways that regulate autophagy, such as the CaMMKβ-activated AMPK pathway and the PKCθ pathway [77,78]. However, in the pathogenesis of pancreatitis, elevated Ca^2+^ concentration can contribute to induce premature activation of zymogens and to increase oxidative stress and mitochondrial dysfunction [98], which may induce autophagy impairment.

#### 2.4.3. Mitochondrial Dysfunction

Ca^2+^ overload leads to mitochondrial dysfunction in pancreatic acinar cells [96]. Mitochondrial dysfunction is one of the prominent hallmarks of early-stage acute pancreatitis [21]. Ca^2+^ overload in the mitochondrial matrix causes a persistent opening of mitochondrial permeability transition pores (MPTPs), which function as nonspecific mitochondrial membrane channels [23,99]. Persistent MPTP opening causes a depolarization of the mitochondrial membrane and a drop in ATP levels, which, in turn, leads to mitochondrial dysfunction [99]. Reduced ATP generation can initiate autophagy through ATP-sensing and AMPK activation. In addition to Ca^2+^ overload, MPTP opening can be induced by a Ca^2+^-independent pathway, which involves reduced ATP synthase activity [99]. However, the accumulation of damaged mitochondria is a major consequence of impaired autophagy in pancreatitis [89]. It has been reported that the inability to remove damaged mitochondria due to impaired autophagy plays an important role in pancreatitis [97].

#### 2.4.4. Inflammation

The severity of acute pancreatitis is largely dependent on the inflammatory responses that occur during the initial stage of the disease [31]. If persistent, inflammation can lead to chronic pancreatitis and to pancreatic ductal adenocarcinoma [30]. Damage to pancreatic acinar cells is caused by genetic factors as well as extrinsic factors, such as alcohol abuse, biliary stones, and infection, which stimulate ROS production in PACs [31]. Injured acinar cells release damage-associated molecular patterns (DAMPs), which are recognized by immune cell receptors. Activated neutrophils and monocytes are recruited to the pancreas to initiate the inflammatory response [31]. Inflammatory mediators, including tumor necrosis factor α (TNF-α), interleukin 1β (IL-1β), IL-6, IL-8, platelet-activating factor (PAF), and chemokines, mediate the cytokine storm and the ensuing inflammatory responses [30]. In particular, the oxidant-sensitive transcription factor NF-κB is rapidly activated at the early stage of acute pancreatitis [100]. Activated by inflammatory cytokines such as TNF-α, IκB is phosphorylated by IκB kinase (IKK), resulting in the release of NF-κB for translocation to the nucleus, where it regulates cytokine gene transcription [100].

Autophagy assists in the inflammation-mediated defense against pathogens through the recruitment of immune cells and by pathogen clearance via autophagosome-mediated engulfment and autophagolysosome-mediated degradation [101]. Autophagy also removes damaged mitochondria to limit inflammasome formation and eliminates adaptor proteins that promote NF-κB activation [91,92]. Therefore, autophagy blockade or impairment contributes to pancreatitis due to loss of function in attenuating inflammation. Since autophagic vacuoles are accumulated in PACs in pancreatitis [68], dysregulated autophagy may occur in PACs in the pathogenesis of pancreatitis.

#### 2.4.5. Lysosomal Dysfunction

In addition to mitochondrial dysfunction, lysosomal dysfunction also occurs in pancreatitis [81,102]. Because of the central role that lysosomes play in the autophagic process, dysregulation of the lysosome function contributes to impaired autophagy. During pancreatitis, autophagic flux is reduced as a result of failed autophagolysosome fusion or lysosomal-mediated cargo degradation [82,88], stemming from insufficient amounts of lysosomal membrane proteins LAMP-1 and -2 and from altered cathepsin processing, respectively [15,80].

## 3. Lycopene and Autophagy

The effect of lycopene on the signaling pathways that modulate autophagy is summarized in Table 1. Treatment with lycopene has a limitation in in-vitro cell culture experiments since lycopene is difficult to solubilize in the cell culture medium. Studies have shown that lycopene reduced cell death by reducing stimulant-induced activation of autophagy by reducing Akt1 and MAPK1 signaling in hippocampal cells [103] and by increasing redox signaling in endothelial progenitor cells [104], kidneys of rats with nephropathy [35,105], and rat hepatocytes [101]. However, lycopene reduced cell death by increasing AMPK-mediated autophagy in hypoxia/reoxygenation-induced H9C2 myocardioblast cells [106]. Even though the effect of lycopene on reducing cell death is different, either inhibiting or enhancing autophagy depending on the experimental conditions, it is evident that lycopene shows a beneficial effect on experimentally–induced cell death. Further studies are necessary to determine the bioavailability of lycopene in the cells after using lycopene in the culture medium in experiments utilizing various cells and tissues.

### 3.1. Inhibition of Cell Death by Suppressing Autophagy

Because autophagic dysfunction is commonly observed in neurodegenerative diseases, the autophagy pathway is viewed as a promising therapeutic target. In cadmium (Cd)-induced hippocampal dysfunction in experimental mouse and cell models, Cd exposure triggered redox stress in hippocampal cells, as antioxidant enzyme activities were decreased while oxidative stresses were promoted. Cd exposure increased the gene expression of the autophagy-related gene (ATG), with increased Ca^2+^ concentration in the hippocampus. All the hippocampal dysfunctions upon Cd exposure were reversed by lycopene treatment to normal situations in mice and in the mouse hippocampal neuronal cell line TH22 [103].

Lycopene treatment of isolated endothelial progenitor cells from rats with type 2 diabetes mellitus (DM) induced with advanced glycation end products (AGEs) resulted in a reduction in apoptotic cell death and oxidative autophagy. The reduction in cell death is attributed to the antioxidant properties of lycopene [104]. Lycopene also plays a beneficial role in the stabilization of mitochondrial membrane potential and the protection of mitochondria by reducing ROS. It has been suggested that lycopene may protect AGE-induced oxidative autophagy in endothelial progenitor cells from patients with DM and offers a new therapy for DM vascular complications.

A study of lycopene amelioration in contrast medium-induced nephropathy in a rat model revealed its antioxidant, anti-inflammatory, antiapoptotic, and antiautophagic effects [35]. Owing to the antioxidant activity of lycopene, the activities of the redox signaling enzymes superoxide dismutase (SOD), catalase (CAT), and glutathione peroxidase (GSH-Px) were decreased, and GSH levels were increased. In addition, lycopene reduced the levels of lipid peroxidation indicator malondialdehyde (MDA) and inflammatory inducible nitrogen oxide synthase (iNOS). iNOS is an important factor for nonspecific host defense. However, a large amount of nitric oxide (NO) generated by iNOS is cytotoxic and inflammatory, and it reacts with superoxide to generate highly toxic peroxynitrite.

In gentamicin-induced nephrotoxicity in rats, pretreatment with lycopene, in combination with rosmarinic acid, countered renal cortical oxidative stress, apoptosis, and autophagy in the kidneys by reducing iNOS levels and increasing GSH, GSH-Px, and SOD levels [105]. In iron-induced oxidative damage in rats, lycopene reduced iron-catalyzed lipid peroxidation by decreasing MDA levels in the liver and colon and enhancing the total SOD activities in serum and tissues [106]. Lycopene reduced iron-induced accumulation of hepatic lysosomes, which triggered autophagy, as determined by the formation of autophagic vesicles in hepatocytes. Thus, lycopene pretreatment prevents iron-induced oxidative stress and pathological autophagy.

### 3.2. Inhibition of Cell Death by Activating Autophagy

Although hypoxia, ROS generation, and ER stress can contribute to autophagy initiation as a protective mechanism, excessive stress signals induce mitochondria-dependent apoptotic cell death. Lycopene prevented the apoptotic cell death of myocardioblast cells caused by hypoxia/reoxygenation by activating autophagy in an AMPK-dependent manner [54].

## 4. Lycopene and Pancreatitis

Several experimental models are widely used to carry out acute pancreatitis research [107]. Cerulein, L-arginine, or bile acids are administered to mouse/rat models (or appropriate cell lines) to induce pancreatitis [108]. Cerulein increases proteolytic enzyme secretion to a level that causes acinar cell autolysis. Necrotizing pancreatitis is experimentally induced by the administration of L-arginine, which induces an increase in free radicals, NO, and inflammatory mediators [108]. Gallstones are one of the common etiologies of pancreatitis. Obstruction of pancreatic flow by gallstones increases the concentration of bile acids, which in turn induce pancreatitis through Ca^2+^ signaling [109].

The effect of lycopene on signaling pathways in experimental pancreatitis is summarized in Table 2 and is discussed below.

Most studies have reported that the antioxidant and anti-inflammatory properties of lycopene mediate its protective effect on pancreatitis. In experimental pancreatitis, cerulein binds to CCK receptors and transiently increases the levels of intracellular Ca^2+^. Increased Ca^2+^ is responsible for the activation of NADPH oxidase, which activates Rac and produces ROS. Increased ROS and inflammation induce the NF-κB signaling pathway and the expression of the inflammatory cytokines IL-1β, IL-6, and TNF-α [25]. Owing to its antioxidative property, lycopene can suppress NF-κB activation and inhibit the inflammatory cytokine IL-6 in pancreatic acinar cells [25,109].

In an acute pancreatitis rat model induced with L-arginine, lycopene administration significantly reduced inflammatory mediators such as TNF-α and downregulated iNOS expression [110]. As discussed above, NO generated by iNOS has a cytotoxic and inflammatory effect, and overproduction of NO in acute pancreatitis has been suggested to be detrimental [110]. Lycopene administration also reduced ROS generation and enhanced GSH synthesis. GSH, the major antioxidant in mammalian cells, is usually depleted in acute pancreatitis, and this is thought to contribute to the severity of the disease [113]. Furthermore, the activity of myeloperoxidase (MPO), an enzyme that stimulates neutrophil infiltration, is reduced by lycopene. Thus, the anti-inflammatory and antioxidant effects of lycopene can attenuate the symptoms of acute pancreatitis.

In other rat models treated with sodium taurocholate, and in rat acinar cells treated with cerulein, lycopene administration decreased the production of the inflammatory mediator cyclooxygenase-2 (COX-2) and NF-κB p65 in the same manner [111]. Lycopene also reduced the levels of the proinflammatory cytokines TNF-α, IL-6, MIP-1α, and MCP-1 and decreased the level of myeloperoxidase (MPO). As an antioxidant, lycopene enhanced the activation of SOD, a ROS scavenger. Researchers suggested that the protective effect of lycopene results from the inhibition of necrotic cell death via the JNK–caspase-3 axis. Necrosis is an important pathogenic mechanism associated with acute pancreatitis. Blocking the initial stage of inflammation and the generation of oxidative stress, however, can attenuate impaired autophagy in acute pancreatitis.

In another study carried out with cerulein-induced acute pancreatitis rat models, lycopene increased GSH levels and decreased the levels of MPO and inflammatory cytokines such as TNF-α and IL-1β in pancreatic tissues [112]. Additionally, lycopene increased Na^+^/K^+^-ATPase activity and reduced malondialdehyde (MDA) levels. To summarize, lycopene inhibits oxidative stress-induced tissue damage and inflammation of pancreatic tissues in experimental pancreatitis models.

## 5. Lycopene, Autophagy, and Pancreatitis

In this review, we have summarized the relationship between impaired autophagy and pancreatitis. Even though lycopene inhibits cell death by reducing or enhancing autophagy in various cells and tissues, studies investigating the effect of lycopene on impaired autophagy related to pancreatitis have not been established. As mentioned above, the effect of lycopene on reducing cell death is different, either inhibiting or enhancing autophagy, depending on the experimental conditions.

**Figure 1:**
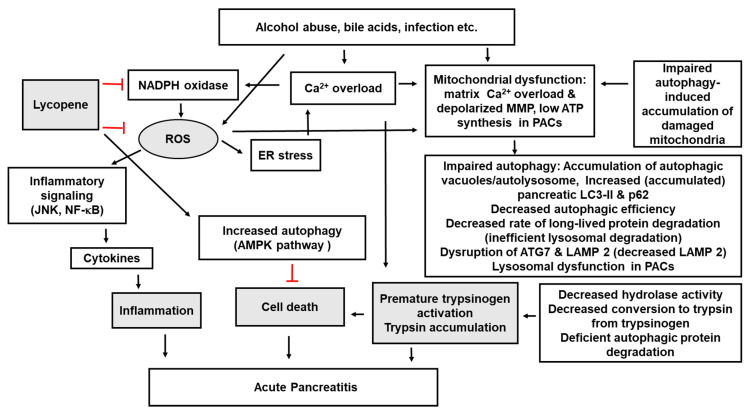
Possible mechanisms by which lycopene attenuates the pathology of acute pancreatitis via its effect on autophagy. Various stimuli (alcohol abuse, bile acids due to duct obstruction by gallstones, infection) lead to increased reactive oxygen species (ROS), intracellular and mitochondrial Ca^2+^ overload, and dysfunction of mitochondria in pancreatic acinar cells (PACs). Activated Ca^2+^ signaling also stimulates ROS production in PACs. Therefore, ROS may be the critical signaling factor to induce pancreatitis. ROS induce mitochondrial dysfunction, as determined by mitochondrial matrix Ca^2+^ overload, reduced mitochondrial membrane potential, and low ATP synthesis in PACs. Mitochondrial dysfunction also can be caused by impaired autophagy, with an accumulation of damaged mitochondria. Mitochondrial dysfunction leads to impaired and dysregulated autophagy, including accumulation of autophagic vacuoles/autolysosomes, increased (accumulated) pancreatic LC3-II and p62, decreased autophagic efficiency, decreased rate of long-lived protein degradation (inefficient lysosomal degradation), disruption of ATG7 and LAMP-2 (decreased LAMP-2), and lysosomal dysfunction in PACs. Decreased hydrolase activity, decreased conversion to trypsin from trypsinogen, and deficient autophagic protein degradation are also shown in acute pancreatitis. All of these events result in the premature activation of trypsinogen and trypsin accumulation in PACs, which are the symptoms of acute pancreatitis. Ca^2+^ overload can directly induce premature activation of trypsinogen. ROS induce ER stress, which increases Ca signaling that may activate nicotinamide adenine dinucleotide phosphate (NADPH) oxidase to produce ROS in PACs. ROS activate inflammatory signaling (JNK, NF-kB) to induce cytokine expression, leading to pancreatic inflammation. Lycopene inhibits NADPH oxidase activity and thus, reduces ROS levels and/or directly scavenges ROS in stimulated PACs. Lycopene prevents ROS- and Ca^2+^-induced mitochondrial dysfunction and impaired autophagy. Lycopene can activate AMPK-dependent autophagy, which inhibits cell death. Therefore, lycopene prevents impaired autophagy, which is shown in acute pancreatitis. Therefore, lycopene may potentially serve as a preventive and therapeutic agent for pancreatitis. Red lines: inhibition. AMPK, 5’-AMP-activated protein kinase; ATG, autophagy-related gene; ER stress, endoplasmic reticulum stress; JNK, c-Jun NH2-terminal kinase; LAMP, lysosomal-associated membrane protein; LC3, microtubule-associated 1A/1B-light chain 3; NADPH, nicotinamide adenine dinucleotide phosphate; NF-κB, nuclear factor-κB; PACs, pancreatic acinar cells; ROS, reactive oxygen species.

Although further work is needed to completely understand the mechanisms by which lycopene confers therapeutic effects via the modulation of autophagy, what is presently known provides significant insight. The possible mechanisms by which lycopene attenuates the pathology of pancreatitis via its effects on autophagy are summarized in Figure 1. In general, the therapeutic effect of lycopene is based on its ability as an antioxidant to suppress ROS signaling and inflammatory responses. Pathogenic factors for pancreatitis such as alcohol, bile acids, and infection increase ROS and Ca^2+^ overload in PACs. Activated Ca^2+^ signaling also stimulates ROS production in PACs. Therefore, ROS may be an important signaling factor to mediate acute pancreatitis. ROS induce mitochondrial dysfunction, as evidenced by mitochondrial matrix Ca^2+^ overload, reduced mitochondrial membrane potential, and low ATP synthesis in PACs. Mitochondrial dysfunction can also be caused by impaired autophagy, with an accumulation of damaged mitochondria. Mitochondrial dysfunction leads to impaired and dysregulated autophagy, including the accumulation of autophagic vacuoles/autolysosomes, increased pancreatic LC3-II and p62, decreased autophagic efficiency, decreased rate of long-lived protein degradation (inefficient lysosomal degradation), disruption of ATG7 and LAMP-2, and lysosomal dysfunction in PACs. Decreased hydrolase activity, decreased conversion to trypsin from trypsinogen, and deficient autophagic protein degradation are also shown in pancreatitis. All these events lead to premature activation of trypsinogen and trypsin accumulation in PACs, which are the symptoms of acute pancreatitis. Ca^2+^ overload can directly induce premature activation of trypsinogen. ROS induce ER stress, which increases Ca^2+^ signaling, which, in turn, activates NADPH oxidase to produce ROS in PACs. ROS activate inflammatory signaling (JNK, NF-κB) to induce cytokine expression, resulting in pancreatic inflammation. Lycopene inhibits NADPH oxidase activity, reduces ROS levels, and/or directly scavenges ROS and thus prevents ROS- and Ca^2+^-induced mitochondrial dysfunction and impaired autophagy. Lycopene can increase antioxidant enzymes and activate AMPK-dependent autophagy, resulting in an inhibition of cell death. Taken together, we propose that lycopene prevents impaired autophagy, which is shown in acute pancreatitis. Consumption of lycopene-rich foods may prevent the development of acute pancreatitis. More studies should be performed to establish the direct effects of lycopene on autophagy signaling molecules using experimental pancreatitis models.

Current research on lycopene, dysregulated autophagy, and pancreatitis is focused on acute pancreatitis because impaired autophagy is a common feature of the initial stage of the disease. Even though acute pancreatitis, recurrent acute pancreatitis, and chronic pancreatitis are known to be part of a continuum, their pathophysiological differences must be defined in order to provide physiological context for understanding the mechanism of autophagy.

## 6. Conclusions and Future Directions

The activity of lycopene in alleviating oxidative stress may attenuate the severity of pancreatitis by preventing impaired autophagy in PACs. Therefore, dietary supplementation with lycopene or consumption of lycopene-rich foods can exert a beneficial effect on impaired autophagy-mediated pancreatitis. Since lycopene may be cleaved by both BCO1 and BCO2, the inhibitory effect of lycopene on the development of pancreatitis may be contributed by intact lycopene and/or its metabolites. More research is clearly needed to identify and characterize additional lycopene metabolites and their biological activities, which will potentially provide valuable insight into the mechanisms underlying the beneficial effects of lycopene in humans, particularly in terms of pancreatitis prevention. In addition, lycopene absorption in humans is in the range of 10–30%, with the remainder being excreted. Therefore, more studies are needed to determine the dose of lycopene needed to prevent pancreatitis. As our understanding of lycopene absorption and metabolism, mode of action for autophagy, and pancreatitis improves, greater insight will be achieved into the role and applications of lycopene and its metabolites in human acute and chronic pancreatitis.

## Figures and Tables

**Table 1:** The effect of lycopene on signaling pathways that modulate autophagy.

Experimental Model	Dose	Signaling Mediators	Notable Results	Ref.
Cadmium-induced hippocampal dysfunction in mice and TH22 cell line	5 mg/kg for mice, 10 μM for cells	Reduced Akt1, MAPK1, signaling	Reduced Cd-induced autophagy (ATG expression) and cell death	[103]
Endothelial progenitor cells isolated from diabetes mellitus rats	10–50 μg/mL	Reduced mitochondrial dysfunction	Reduced apoptosis and oxidative autophagy	[104]
Contrast-induced nephropathy in rats	4 mg/kg	Enhanced antioxidant enzymes	Reduced cell death	[35]
Gentamicin-induced nephrotoxicity in rats	4 mg/kg	Enhanced SOD, GSH	Reduced oxidative stress-induced apoptosis and autophagy	[105]
Iron-induced oxidative damage in rats	10 mg/kg, 15 mg/kg, 20 mg/kg	Reduced formation of autophagic vesicles	Inhibited oxidative stress, and pathologic autophagy	[106]
Hypoxia/reoxygenation-induced H9C2 myocardioblast cells	2.5 μM, 5 μM	Increased AMPK activity	Reduced apoptotic cell death through increased autophagy	[54]

**Table 2:** The effect of lycopene on signaling pathways in pancreatitis pathology.

Experimental Model	Dose	Signaling Mediators	Notable Results	Ref.
Cerulein-induced rats pancreatic acinar cells	2 μmol/L, 5 μmol/L	Reduced NF-κB activity	Decreased cytotoxicity	[109]
L-arginine-induced acute pancreatitis in rats	50 mg/kg	Reduced TNF-β and increased GSH	Pancreatitis amelioration	[110]
Sodium taurocholate-induced severe acute pancreatitis in rats	10 mg/kg	Reduced NF-κB p65 activity	Pancreatitis amelioration	[111]
Cerulein-stimulated rats pancreatic acinar cells	2 μmol/L, 10 μmol/L	Blocked JNK–caspase-3 axis	Decreased cytotoxicity	[112]

## Abbreviations

AGE	Advanced glycation end product
ATG	Autophagy-related gene
Bcl-2	B-cell lymphoma 2
BCO1	β-Carotene oxygenase 1; β -Carotene 15,15′-oxygenase
BCO2	β-Carotene oxygenase 2; β-Carotene 9′,10′-oxygenase
CaMKKβ	Calmodulin-dependent kinase kinase-β
CatB	Cathepsin B
CAT	Catalase
CCK	Cholecystokinin
CD36	Cluster determinant 36
COX	Cyclooxygenase
DAMP	Damage-associated molecular patterns
DM	Diabetes mellitus
ER	Endoplasmic reticulum
ERK	Extracellular signal-regulated kinase
FoxO	Forkhead box O
GSH	Glutathione
GSH-Px	Glutathione peroxidase
GSK3β	Glycogen synthase kinase 3β
IL	Interleukin
iNOS	Inducible nitrogen oxide syntase
IKK	IκB kinase
IRS	Insulin receptor substrate
IκB	Inhibitor of κB
JNK	c-Jun NH2-terminal kinase
LAMP	Lysosomal-associated membrane protein
LC3	Microtubule-associated 1A/1B-light chain 3
LKB1	Liver kinase B1
MAPK	Mitogen-activated protein kinase
MAPKK/MEK	Mitogen-activated protein kinase kinase
MCP	Monocyte chemoattractant protein
MDA	Malondialdehyde
MIP	Macrophage inflammatory protein
MPO	Myeloperoxidase
MPTP	Mitochondrial permeability transition pore
mTORC	Mammalian target of rapamycin complex
NADPH	Nicotinamide adenine dinucleotide phosphate hydrogen
NF-κB	Nuclear factor κ B
NO	Nitric oxide
PACs	Pancreatic acinar cells
PAF	Platelet-activating factor
PDK	Phosphoinositide-dependent protein kinase 1
PIP3	Phosphatidylinositol (3,4,5)-trisphosphate
PKB	Protein kinase B
PKC	Protein kinase C
PtdIns3KC3	Class III phosphatidylinositol 3-kinase
Raf	Rapidly accelerated fibrosarcoma
Rag	Ras-related GTP binding protein
Ras	Retrovirus-associated DNA sequences
RB1CC1/FIP200	RB1-inducible coiled-coil 1/Focal adhesion kinase family-interacting protein of 200 kDa
Rheb	Ras homolog enriched in brain
ROS	Reactive oxygen species
SNARE	soluble N-ethylmaleimide-sensitive factor attachment protein receptors
SOD	Superoxide dismutase
SRB	Scavenger receptor class B
SR-B1	Scavenger receptor class B type-1
TNF	Tumor necrosis factor
TSC	Tuberous sclerosis
ULK	Unc-51-like kinases
V-ATPase	Vacuolar ATPase
Vps34	Vacuolar protein sorting 34
